# Supplementary material for: Modern-like deep water circulation in Indian Ocean caused by Central American Seaway closure
Source: Nat Commun. 2022 Dec 8;13:7561. doi: 10.1038/s41467-022-35145-0 (PMC9729181; doi:10.1038/s41467-022-35145-0)
Supplement: Supplementary file 1 — Supplementary Information [file 41467_2022_35145_MOESM1_ESM.pdf]

Supplementary Information

Modern-like deep water circulation in Indian Ocean caused by Central American Seaway closure

*Priyesh Prabhat[1,2], *Waliur Rahaman[1], Nambiyathodi Lathika[1], Mohd Tarique[1], Ravi Mishra[1] and Meloth Thamban[1]

[1]National Centre For Polar And Ocean Research, Ministry of Earth Science, Goa, India.

[2]School of Earth, Ocean and Atmospheric Sciences, Goa University, Goa, India

*Corresponding author: priyeshprabhat@gmail.com, waliur@ncpor.res.in

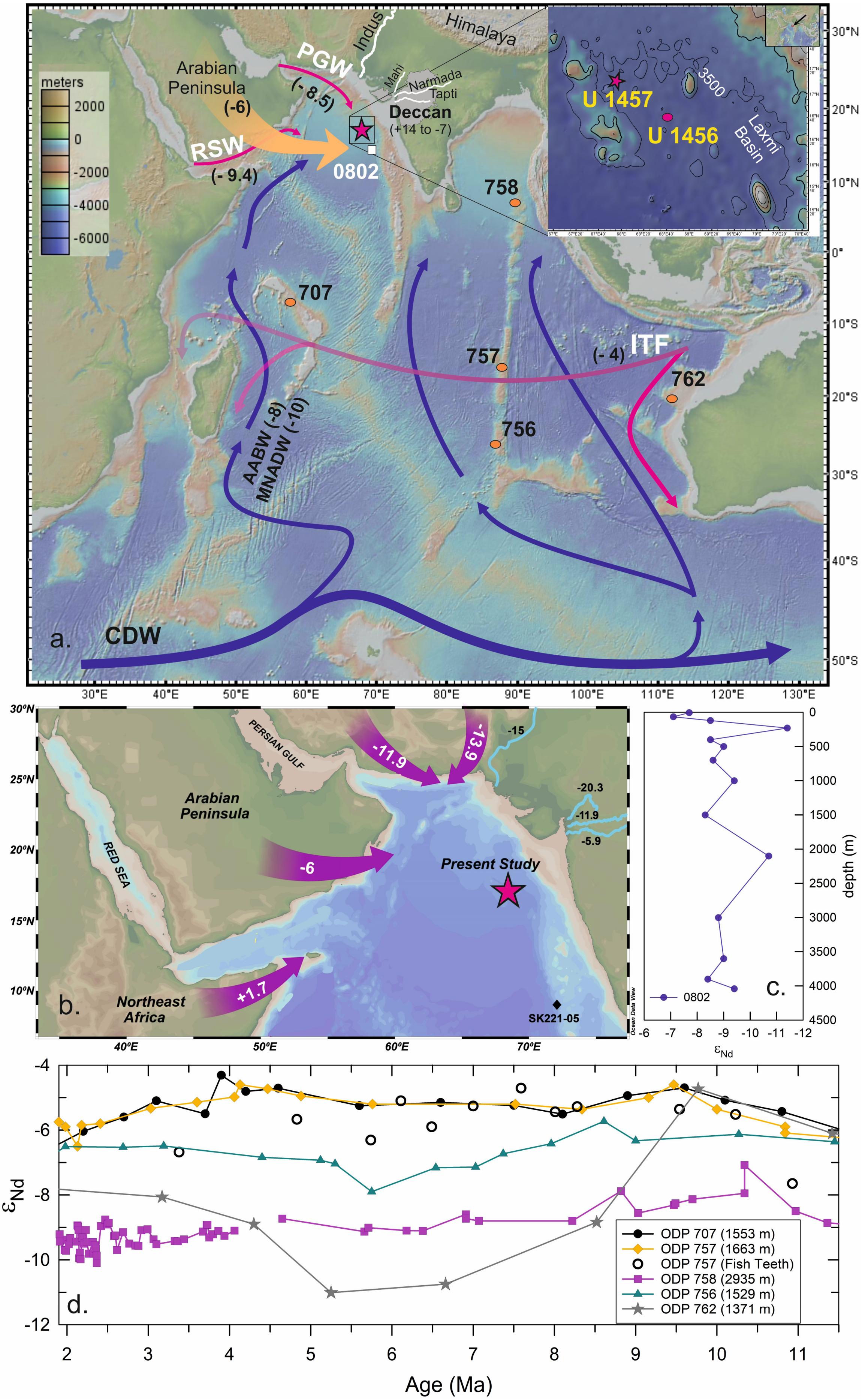
meters
2000
0
-2000
-4000
-6000
Arabian Peninsula
(-6)
PGW
(- 8.5)
Indus
Himalaya
Mahi
Narmada
Tapti
Deccan
(+14 to -7)
RSW
(- 9.4)
0802
758
707
757
(- 4)
ITF
762
756
AABW (-8)
MNADW (-10)
CDW
a.
U 1457
U 1456
3500
Laxmi Basin
PERSIAN GULF
-11.9
-13.9
-15
-20.3
-11.9
-5.9
Arabian Peninsula
RED SEA
-6
Present Study
+1.7
Northeast Africa
b.
SK221-05
0802
c.
depth (m)
d.
Age (Ma)
ODP 707 (1553 m)
ODP 757 (1663 m)
ODP 757 (Fish Teeth)
ODP 758 (2935 m)
ODP 756 (1529 m)
ODP 762 (1371 m)

Supplementary Fig. 1. Potential sources of Nd with their characteristic ε_{Nd} values and authigenic ε_{Nd} records from the Indian Ocean. (a). Location map with the water mass pathways in the Indian Ocean. The pink star marks the present study's location (IODP 355 U1457), while the orange circles indicate existing ε_{Nd} records from the Indian Ocean for the studied time interval (ODP sites 707[1], 758[1], 757[1,2], 756[3], 762[3]). The white square marks the proximal seawater station 0802[4], whose value has been used for the modern ε_{Nd} values. Surface water masses are indicated by pink arrows, whereas blue arrows indicate deep water masses. Orange arrow marks the aeolian dust influx from the Arabian Peninsula. ε_{Nd} value of different water masses (given in bracket). PGW- Persian Gulf Water, ITF- Indonesian Throughflow Water, RSW- Red Sea Water, CDW- Circumpolar Deep Water, MNADW- Modified North Atlantic deep water, AABW- Antarctic Bottom Water, ASW- Arabian Sea Water (which includes PGW, RSW and Arabian Sea high salinity water). The image in the inset shows the high-resolution bathymetry of the core location. The base map was produced using online Geomap app (http://www.geomapapp.org); (b) Orange arrow marks the aeolian dust influx from the nearby continents with their ε_{Nd} values given in white[5,6] (Arabian Peninsula -6, Northeast Africa +1.7, Persia -11.9, Thar desert -13.9) along with the rivers ε_{Nd} values which contributes to study site[4]. The base map in the Panel b was produced using Ocean Data View Software (https://odv.awi.de/); (c) Modern seawater column ε_{Nd} profile at station 0802[4]; (d) Published authigenic ε_{Nd} records from Indian Ocean ODP site 707, 757 and 758[1,7], 756, 762[3], black open circle represents fish teeth record from ODP 707[2].

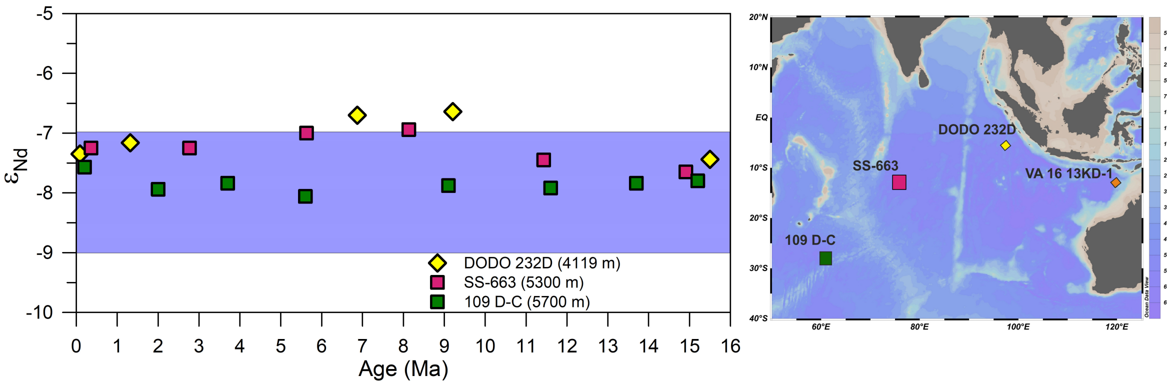

Supplementary Fig. 2. Deepwater Fe-Mn crust ε_{Nd} records[8,9] from the Indian Ocean and their locations. Blue shading represents the modern day ε_{Nd} value of the AABW.

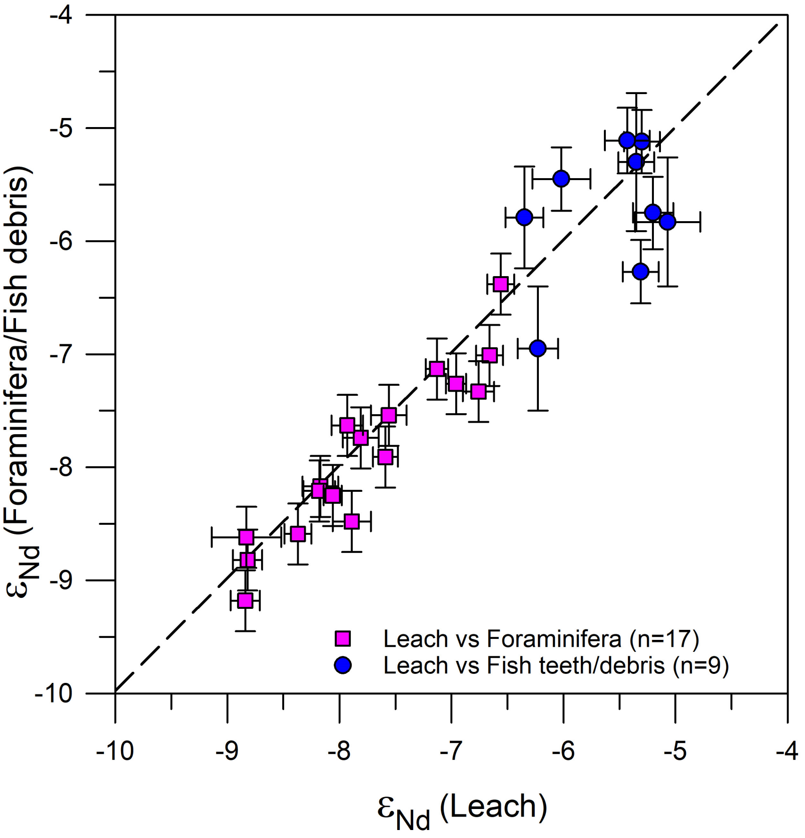

Supplementary Fig. 3. Assessing fidelity of authigenic ε_{Nd} records. Cross plot of the ε_{Nd} of leach versus corresponding ε_{Nd} fish teeth/debris (present study) and ε_{Nd} foraminifera published record from Lathika, et al. [10] and ε_{Nd} of leach vs ε_{Nd} fish teeth/debris (present study). Dashed line represents 1:1 equiline. Error bar represents external error (2σ) of the ε_{Nd} measurements.

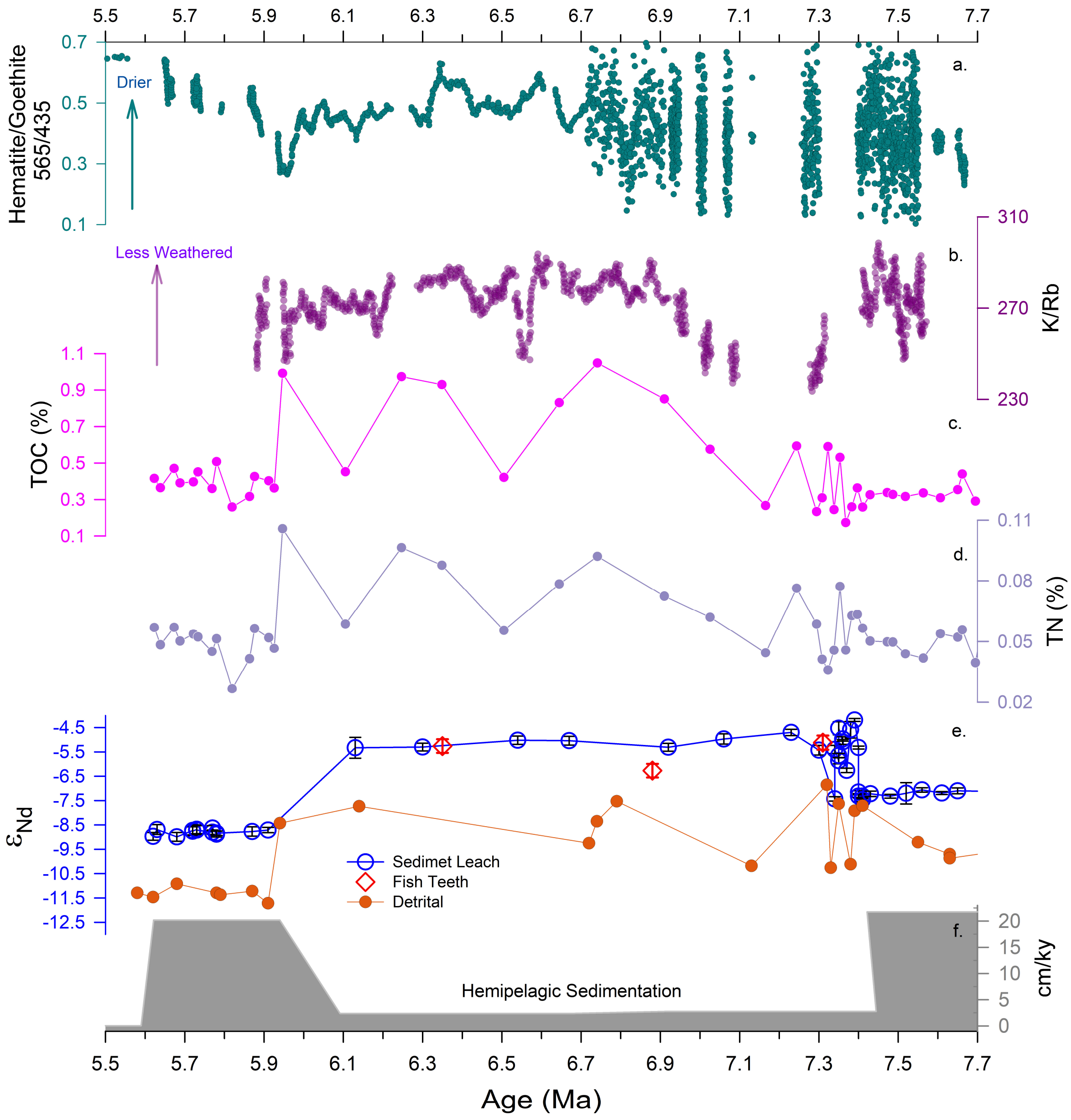

Supplementary Fig. 4. Compilation of various proxy records from the IODP 355 expedition sites. (a) Hematite/goethite ratio from 565/435 reflectance ratio of site U1456[11]; (b) K/Rb ratio as a proxy for chemical weathering for site U1456[11]; (c) Total organic carbon (TOC %) record from site U1457[12]; (d) Total Nitrogen (%) record from site U1457[12]; (e) authigenic and detrital ε_{Nd} record from site U1457; (f) sedimentation rate for site U1457[13,14]. Error bar represents external error (2σ) of the ε_{Nd} measurements.

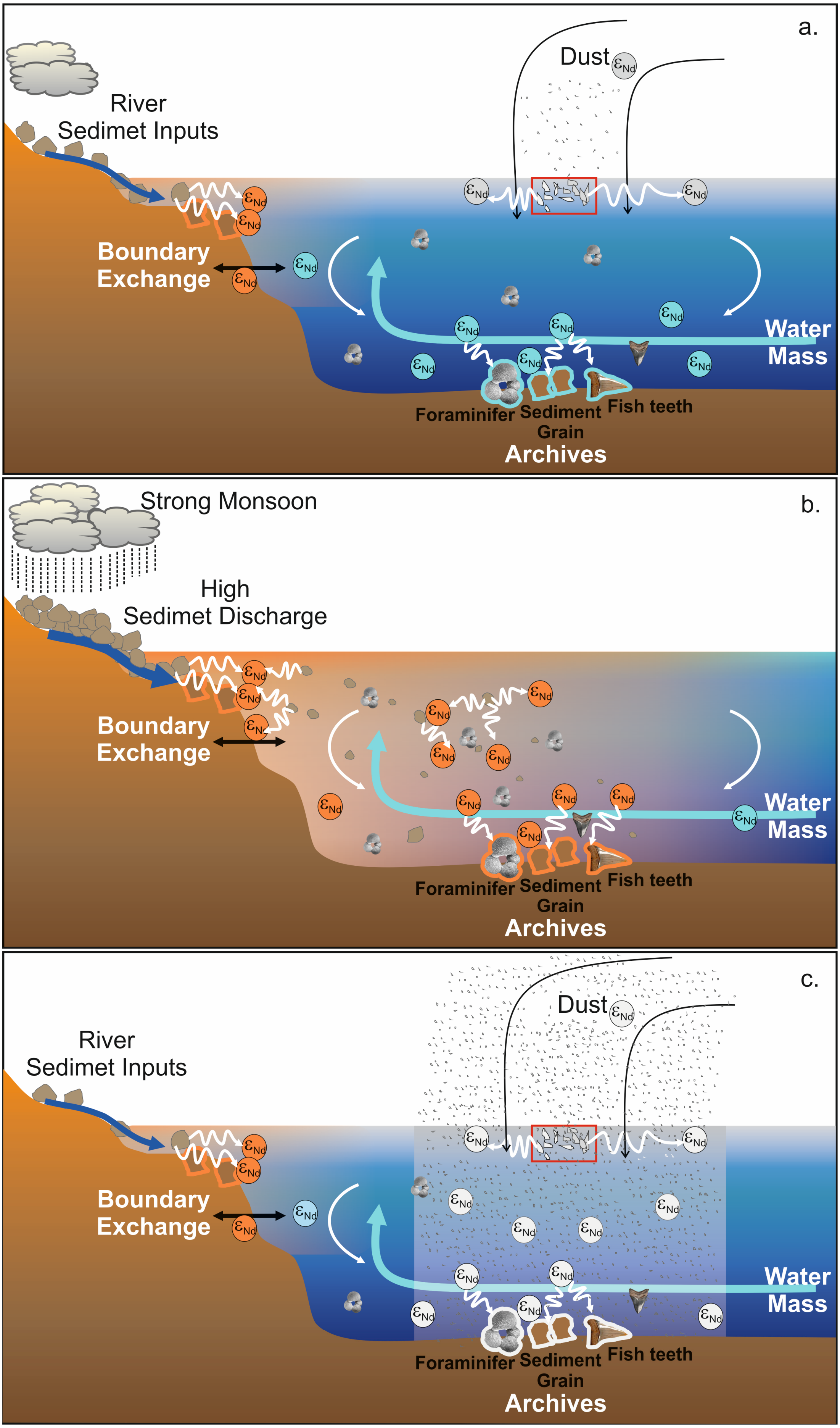
a.
Dust
River
Sedimet Inputs
Boundary
Exchange
Water
Mass
Foraminifer
Sediment
Grain
Fish teeth
Archives
Strong Monsoon
b.
High
Sedimet Discharge
Boundary
Exchange
Water
Mass
Foraminifer
Sediment
Grain
Fish teeth
Archives
c.
Dust
River
Sedimet Inputs
Boundary
Exchange
Water
Mass
Foraminifer
Sediment
Grain
Fish teeth
Archives

Supplementary Fig. 5. An illustration of how deep water acquires ε_{Nd} values in different scenarios and archived its signature in the authigenic phase. (a) This figure shows how various archives such as authigenic Fe-Mn coatings on the foraminifera, sediment grains and fish teeth/debris deposited in the open ocean acquire deep seawater ε_{Nd} signature. The influence of other processes/sources, such as dissolution/desorption of particulate Nd from the dust deposition (shown by grey tinge) and river-borne sediment are restricted within the top few hundred meters in the seawater column[4] under moderate aeolian and riverine input. The impact of boundary exchange process is restricted to shelf area. (b) During high sediment discharge from rivers due to strong precipitation, river particles may interact with the water column and alter its ε_{Nd} values. This altered ε_{Nd} gets deposited as the authigenic coating of the archives. This scenario has been reported from the BoB[15], while the Arabian Sea is not affected by such process[4,10]; (c) During high eolian dust deposition, dust can alter deep water ε_{Nd} signature through the dissolution and/or diagenetic processes. This altered authigenic values will be recorded in all the archives as shown in the panel. The observed radiogenic excursion in the present study could be attributed to this process. Blue arrow – river inputs, cyan arrow – seawater circulation, curved white arrow – water mass mixing. Orange circle- ε_{Nd} leached from the river sediments to the water column, cyan circle- ε_{Nd} of the water mass, grey circle- ε_{Nd} leached from the dust particles to the water column.

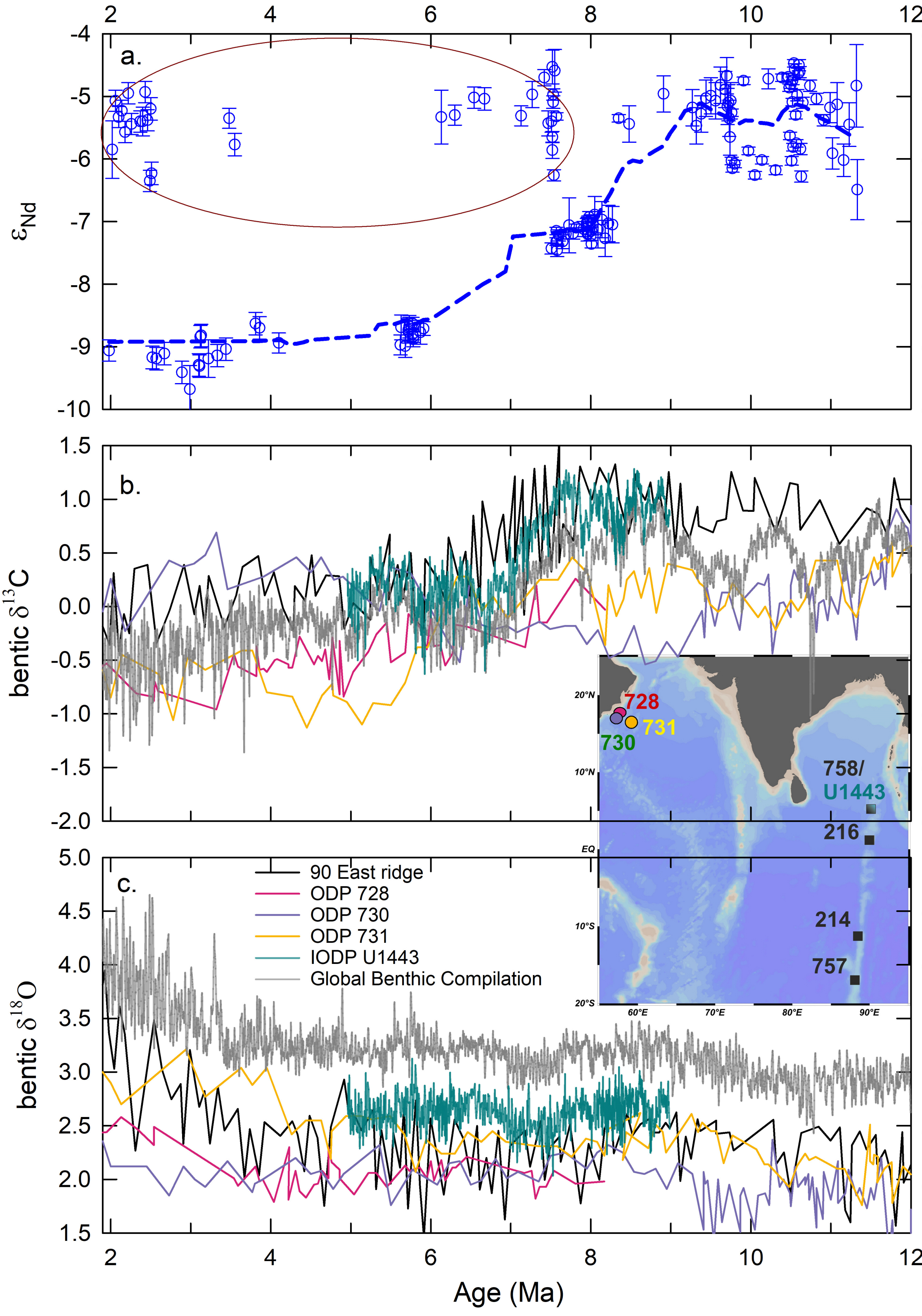

Supplementary Fig. 6. Comparison of the authigenic ε_{Nd} record with the benthic carbon and oxygen isotope records from the Indian Ocean**.** (a) The authigenic ε_{Nd} record from IODP site U1457 (present study); (b) benthic $\delta^{13}C$ isotope record from the Arabian Sea[16,17], 90 East ridge[18,19] in the eastern Indian Ocean, global benthic compilation[20]; (c) benthic $\delta^{18}O$ isotope

record from the Arabian Sea[16,17], 90 East ridge[18,19] in the eastern Indian Ocean, global benthic compilation[20]. Error bar represents external error (2σ) of the ε_{Nd} measurements.

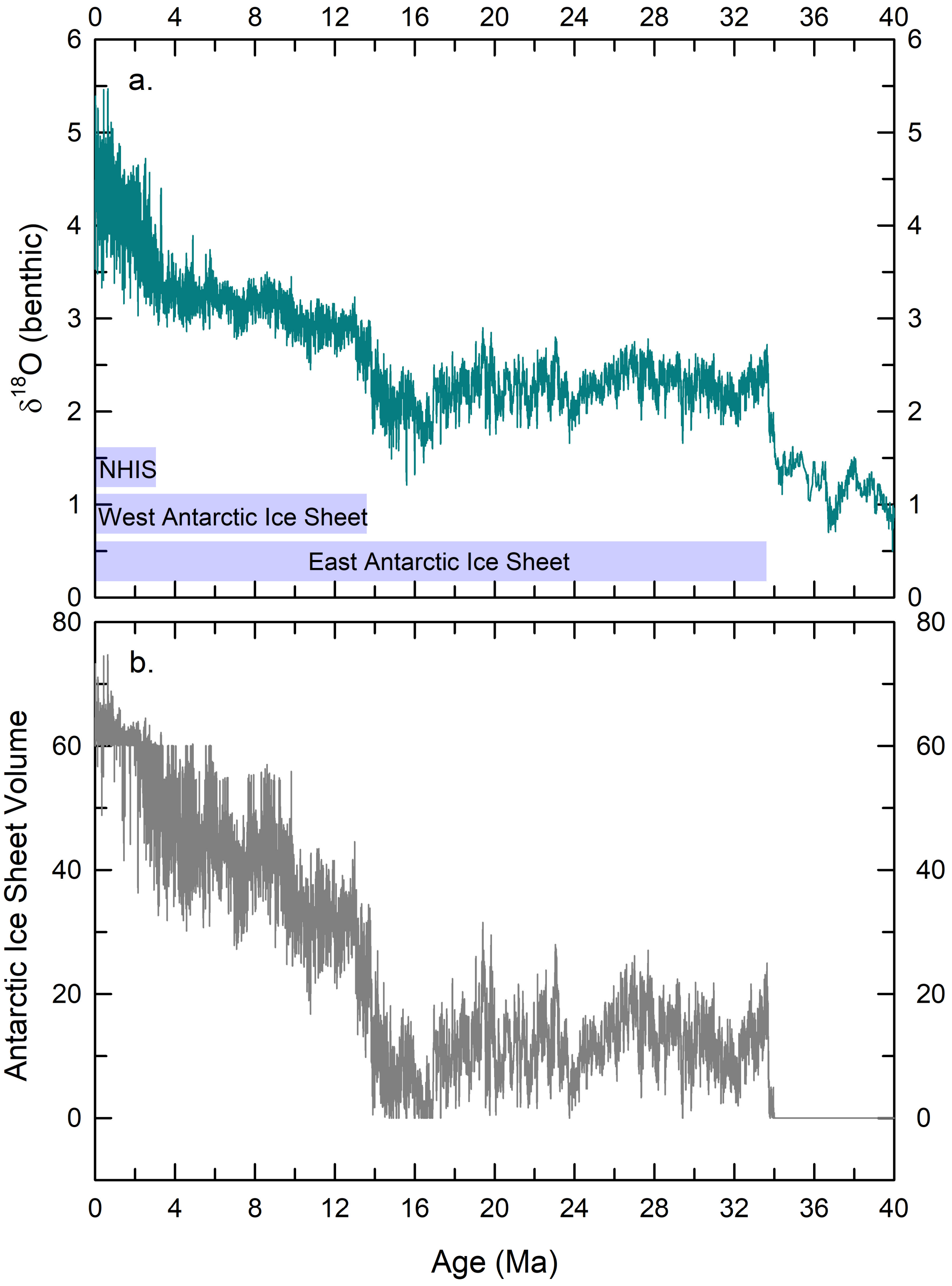

Supplementary Fig. 7. Role of polar ice sheets in the deep water circulation. (a) Benthic $\delta^{18}O$ isotope record[20]; (b) Antarctic ice sheet volume (m sea level equivalent)[21]. NHIS – Northern Hemisphere Ice Sheet.

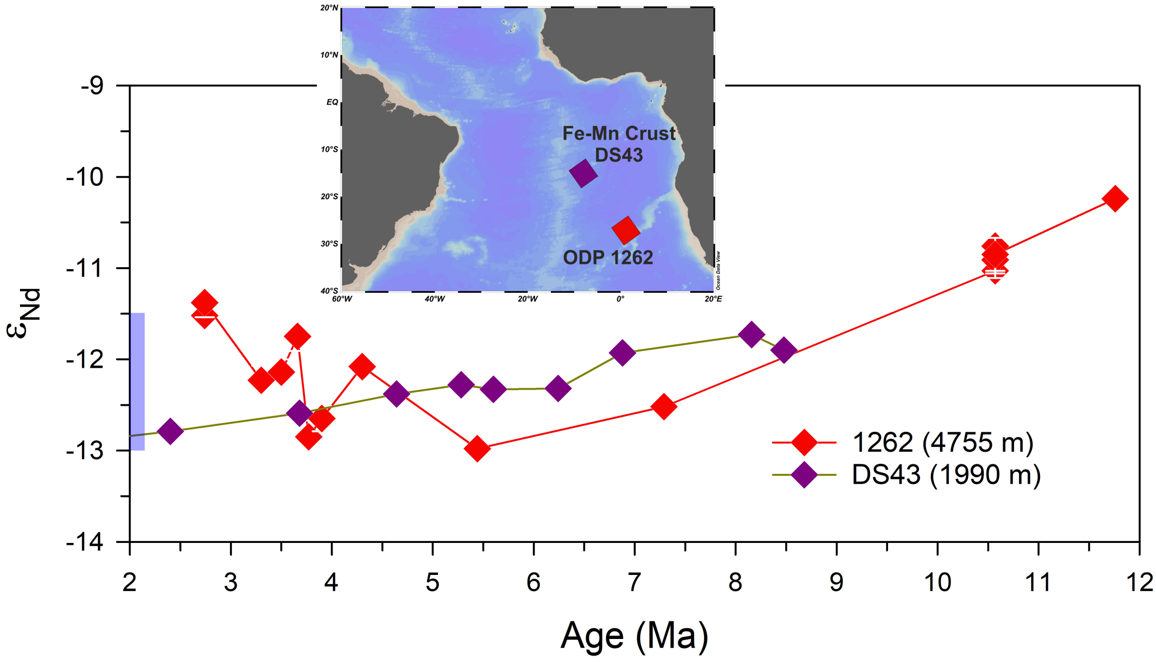

Supplementary Fig. 8. Evolution of northern component of water (NCW) based on the authigenic ε_{Nd} records from Fe-Mn Crust DS43[22] and ODP Site 1262[23]. The blue bar represents the modern ε_{Nd} value of NADW from the Angola basin[24].

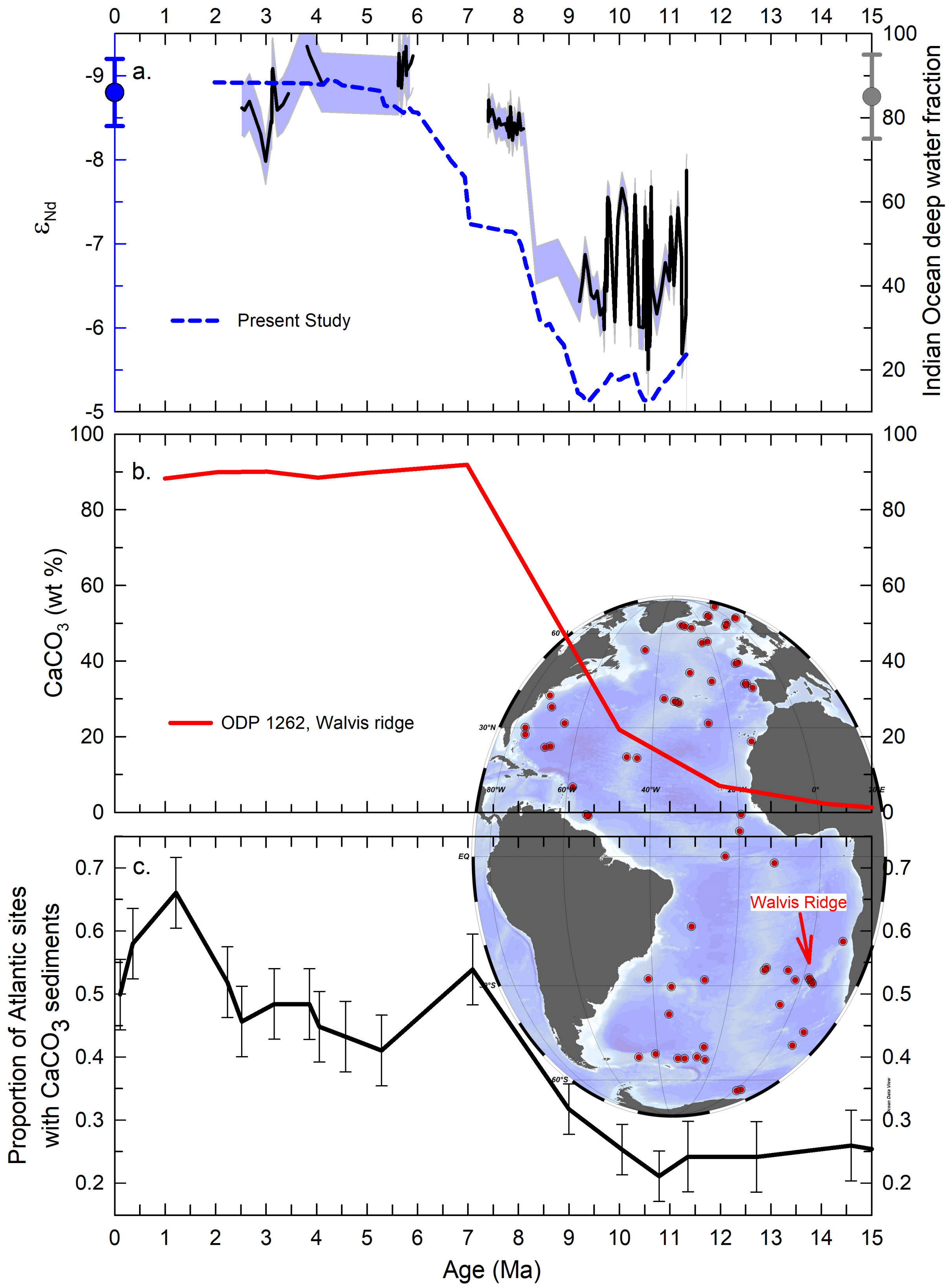

Supplementary Fig. 9. Role of deep water circulation in carbonate chemistry. (a) Authigenic ε_{Nd} record (two-point smoothened, blue line) of site U1457 and its corresponding Indian Ocean deep water fractions (black line with grey error envelope) with their modern-day

values plotted on their respective axis (filled circle); (b) calcium carbonate weight percentage (wt %) for site ODP 1262 (water depth 4769 m) (Zachos et al. 2004); (c) proportion $CaCO_3$ sediments from the DSDP and ODP sites (sites shown in map) in the Atlantic Ocean.

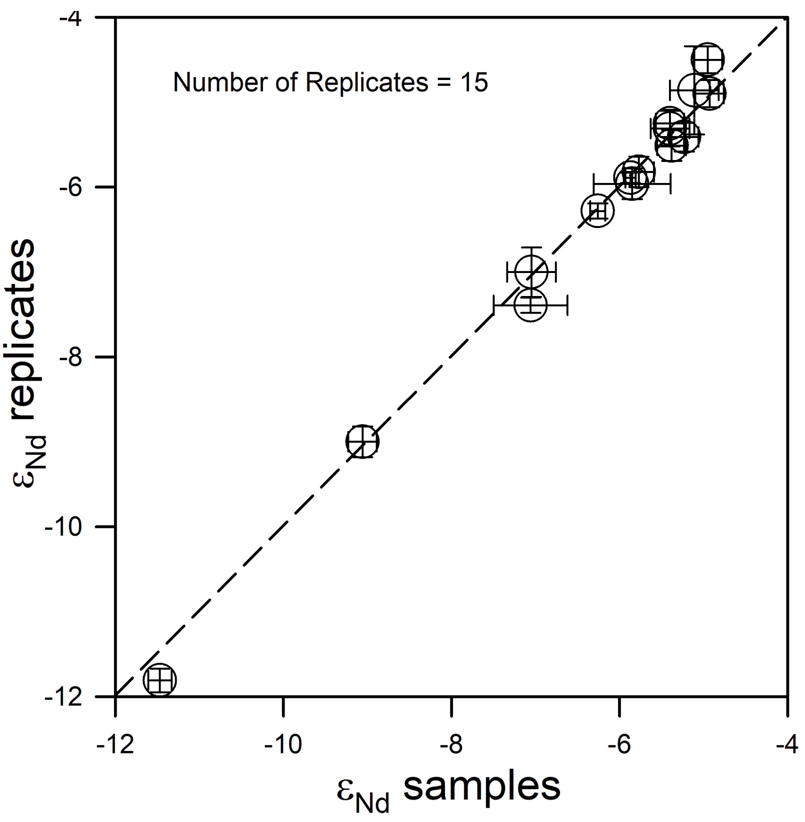

Supplementary Fig. 10. Replicate analysis of Nd isotopes. To ascertain the quality of the analysis, Nd isotopes were measured in the replicates and shown in the cross plot. A total of fifteen replicates were analysed, of which most of the Nd isotope data fall on the equiline (1:1).

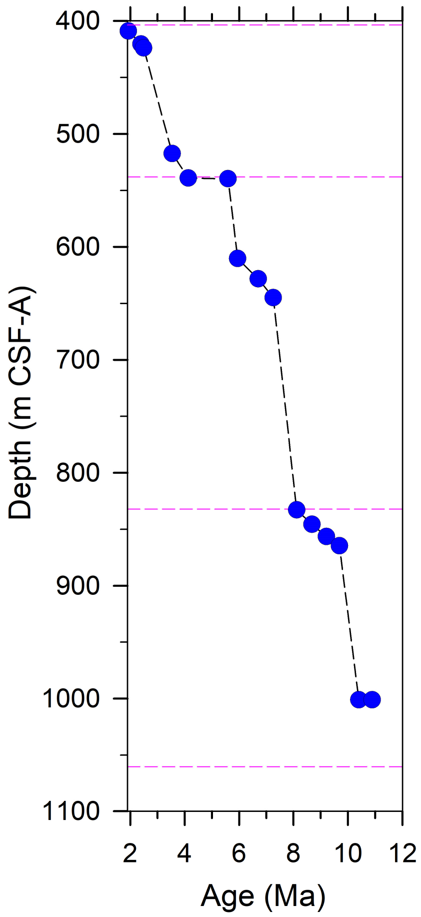

Supplementary Fig. 11. Chronology of core U1457 C. Age vs depth for site U1457 C. Pink lines mark the hiatus. (References are provided in Supplementary Table 1).

Supplementary Table 1: Biostratigraphic datums used for age depth model for the study site U1457 C.

Event	Datum	Age (Ma) Indian Ocean Calibrated	References
LO of	*Discoaster brouweri*	1.93	14
LO of	*Discoaster pentaradiatus*	2.39	14
LO of	*Discoaster surculus*	2.49	13
LO of	*Sphenolithus spp.*	3.54	13
FO of	*Discoaster tamalis*	4.13	13
LO of	*Discoaster quinqueramus*	5.59	14
LO of	*Nicklithus amplificus*	5.94	13
FO of	*Nicklithus amplificus*	6.7	13
FO of	*Amaurolithus spp.*	7.25	13
FO of	*Discoaster quinqueramus*	8.12	14
LO of	*Minylitha convallis*	8.68	14

LO of	*Discoaster bollii*	9.21	14
LO of	*Catinaster coalitus*	9.69	14
FO of	*Catinaster coalitus*	10.89	14

FO- First Occurrence; LO- Last Occurrence; spp.- species

Supplementary Table 2: Neodymium isotopic composition of IODP 355, site U1457 C

CSF-A (m)	Age (Ma)	$^{143}Nd/^{144}Nd$	ε_{Nd}	external error ε_{Nd} (2σ)
Authigenic ε_{Nd}				
405.10	1.98	0.512174	-9.06	0.17
405.10R		0.512177	-9.00	0.18
406.10	2.02	0.512338	-5.85	0.46
406.10R		0.512332	-5.96	0.18
407.10	2.06	0.512378	-5.07	0.17
408.08	2.10	0.512365	-5.33	0.17
409.04	2.14	0.512370	-5.23	0.17
409.04R		0.512361	-5.41	0.17
410.04	2.18	0.512353	-5.57	0.17
411.00	2.22	0.512384	-4.95	0.17
411.00R		0.512407	-4.50	0.16
412.05	2.26	0.512359	-5.44	0.17
414.80	2.37	0.512361	-5.40	0.17
414.80R		0.512369	-5.25	0.16
415.80	2.40	0.512361	-5.40	0.23
415.80R		0.512366	-5.31	0.21
416.82	2.43	0.512385	-4.93	0.17
416.82R		0.512387	-4.90	0.16
417.82	2.46	0.512362	-5.38	0.17
417.82R		0.512356	-5.51	0.18
418.82	2.49	0.512313	-6.35	0.17
419.82	2.50	0.512371	-5.20	0.18
420.83	2.51	0.512318	-6.23	0.18
421.83	2.52	0.512168	-9.17	0.18
425.50	2.57	0.512167	-9.19	0.18
434.98	2.67	0.512171	-9.11	0.18
454.66	2.89	0.512155	-9.41	0.18
463.30	2.99	0.512142	-9.68	0.38
473.00	3.10	0.512162	-9.29	0.18
474.00	3.11	0.512161	-9.31	0.18
475.00	3.12	0.512185	-8.84	0.18
475.69	3.13	0.512186	-8.82	0.18

483.71	3.22	0.512167	-9.19	0.30
493.60	3.33	0.512170	-9.14	0.18
503.10	3.44	0.512175	-9.04	0.18
507.10	3.48	0.512364	-5.35	0.16
512.80	3.55	0.512342	-5.77	0.18
512.80R		0.512340	-5.82	0.18
522.73	3.81	0.512196	-8.63	0.18
531.24	3.86	0.512192	-8.70	0.18
533.20	4.10	0.512180	-8.94	0.16
540.90	5.62	0.512178	-8.97	0.16
541.90	5.63	0.512193	-8.69	0.20
551.70	5.68	0.512177	-8.99	0.18
560.30	5.72	0.512191	-8.73	0.18
561.29	5.72	0.512189	-8.76	0.18
562.31	5.73	0.512193	-8.68	0.18
563.31	5.73	0.512192	-8.71	0.18
570.00	5.77	0.512187	-8.80	0.09
571.00	5.77	0.512196	-8.63	0.10
572.00	5.78	0.512183	-8.88	0.12
573.02	5.78	0.512185	-8.84	0.09
590.40	5.87	0.512188	-8.77	0.19
600.10	5.91	0.512191	-8.71	0.11
609.80	6.13	0.512365	-5.33	0.43
613.81	6.30	0.512366	-5.30	0.16
619.50	6.54	0.512381	-5.02	0.17
622.49	6.67	0.512380	-5.04	0.18
629.20	7.13	0.512366	-5.31	0.16
633.20	7.27	0.512383	-4.97	0.21
638.77	7.42	0.512397	-4.70	0.13
648.60	7.47	0.512360	-5.43	0.20
657.30	7.51	0.512257	-7.43	0.09
658.30	7.51	0.512361	-5.40	0.17
659.25	7.52	0.512338	-5.86	0.13
660.26	7.52	0.512348	-5.65	0.09
661.26	7.52	0.512406	-4.53	0.27
662.26	7.53	0.512377	-5.09	0.10
663.28	7.53	0.512383	-4.97	0.09
664.28	7.54	0.512317	-6.26	0.09
664.28R		0.512316	-6.28	0.09
667.00	7.55	0.512403	-4.59	0.34
670.00	7.57	0.512365	-5.32	0.06
671.00	7.57	0.512271	-7.15	0.06
672.00	7.57	0.512263	-7.32	0.06
672.98	7.58	0.512263	-7.32	0.06

673.65	7.58	0.512255	-7.46	0.10
677.70	7.60	0.512268	-7.22	0.09
687.40	7.65	0.512263	-7.32	0.11
697.14	7.69	0.512269	-7.20	0.08
706.80	7.73	0.512276	-7.06	0.44
706.80R		0.512259	-7.39	0.09
716.49	7.78	0.512269	-7.19	0.09
726.20	7.82	0.512274	-7.10	0.06
745.60	7.91	0.512273	-7.12	0.12
754.30	7.95	0.512278	-7.02	0.07
755.30	7.96	0.512269	-7.20	0.11
756.30	7.96	0.512277	-7.03	0.10
757.3	7.97	0.512279	-7.01	0.10
758.30	7.97	0.512269	-7.19	0.12
759.26	7.98	0.512283	-6.93	0.09
763.96	8.00	0.512280	-6.98	0.09
765.00	8.00	0.512268	-7.23	0.07
765.97	8.01	0.512272	-7.15	0.06
767.00	8.01	0.512261	-7.36	0.06
774.58	8.05	0.512285	-6.89	0.29
784.40	8.09	0.512273	-7.13	0.29
794.10	8.14	0.512281	-6.97	0.29
803.80	8.18	0.512265	-7.27	0.29
813.50	8.22	0.512277	-7.03	0.29
823.20	8.27	0.512277	-7.05	0.29
823.20R		0.512279	-7.00	0.29
832.90	8.35	0.512364	-5.35	0.06
842.58	8.48	0.512359	-5.44	0.29
851.30	8.91	0.512383	-4.96	0.29
852.30	9.27	0.512373	-5.18	0.29
853.30	9.32	0.512357	-5.47	0.29
854.30	9.38	0.512367	-5.28	0.29
855.30	9.44	0.512380	-5.03	0.29
856.29	9.50	0.512382	-4.99	0.29
857.31	9.56	0.512378	-5.07	0.29
858.31	9.62	0.512391	-4.82	0.29
859.28	9.68	0.512387	-4.89	0.29
861.00	9.70	0.512399	-4.67	0.29
861.98	9.71	0.512374	-5.16	0.29
863.00	9.72	0.512377	-5.10	0.29
864.00	9.73	0.512364	-5.35	0.29
865.00	9.74	0.512349	-5.65	0.29
866.00	9.75	0.512378	-5.07	0.29
867.00	9.76	0.512368	-5.26	0.06

868.00	9.76	0.512329	-6.03	0.06
869.00	9.77	0.512323	-6.15	0.06
871.70	9.80	0.512326	-6.08	0.06
884.40	9.91	0.512394	-4.75	0.06
891.10	9.97	0.512337	-5.87	0.06
891.10R		0.512336	-5.89	0.06
900.80	10.05	0.512317	-6.26	0.06
910.50	10.14	0.512330	-6.02	0.06
920.28	10.22	0.512396	-4.72	0.16
929.90	10.31	0.512321	-6.18	0.07
939.60	10.39	0.512397	-4.70	0.06
948.30	10.47	0.512398	-4.69	0.06
949.30	10.48	0.512389	-4.86	0.06
950.30	10.49	0.512350	-5.63	0.06
951.30	10.50	0.512391	-4.81	0.06
952.30	10.51	0.512329	-6.03	0.07
953.30	10.52	0.512394	-4.75	0.06
954.30	10.52	0.512377	-5.09	0.06
955.30	10.53	0.512340	-5.81	0.06
956.30	10.54	0.512409	-4.47	0.06
957.30	10.55	0.512404	-4.57	0.09
958.00	10.56	0.512366	-5.30	0.06
959.00	10.57	0.512343	-5.75	0.06
961.00	10.58	0.512383	-4.98	0.09
962.00	10.59	0.512400	-4.64	0.09
963.00	10.60	0.512408	-4.50	0.09
964.00	10.61	0.512401	-4.61	0.09
965.00	10.62	0.512339	-5.84	0.09
966.00	10.63	0.512316	-6.28	0.09
968.70	10.65	0.512376	-5.10	0.09
978.40	10.74	0.512390	-4.83	0.09
988.10	10.82	0.512379	-5.04	0.09
997.83	10.91	0.512362	-5.38	0.09
1007.20	10.99	0.512372	-5.18	0.24
1011.14	11.02	0.512335	-5.91	0.25
1017.23	11.08	0.512375	-5.13	0.35
1026.50	11.16	0.512329	-6.02	0.26
1034.40	11.23	0.512359	-5.45	0.35
1044.59	11.32	0.512391	-4.83	0.66
1045.43	11.33	0.512305	-6.49	0.48
Fish Teeth/Debris ε_{Nd}				
419.34	2.50	0.512341	-5.79	0.45
419.74	2.50	0.512343	-5.75	0.32
420.34	2.51	0.512282	-6.95	0.55

504.2	3.45	0.512349	-5.64	0.56
506.34	3.47	0.512366	-5.30	0.61
614.8	6.35	0.512375	-5.12	0.28
628.2	7.09	0.512317	-6.27	0.28
650.6	7.48	0.512376	-5.11	0.29
650.6R		0.512389	-4.86	0.52
857.31	9.56	0.512339	-5.83	0.57
1025.89	11.15	0.512359	-5.45	0.28
Detrital ε_{Nd}				
405.1	1.98	0.512024	-11.97	0.14
407.1	2.06	0.512246	-7.64	0.14
413.01	2.30	0.512275	-7.08	0.14
420.83	2.51	0.512193	-8.68	0.14
463.3	2.99	0.512019	-12.08	0.14
512.8	3.55	0.512218	-8.20	0.14
541.9	5.63	0.512050	-11.47	0.14
541.9R		0.512033	-11.81	0.14
600.1	5.91	0.512037	-11.72	0.14
609.8	6.13	0.512241	-7.73	0.14
657.3	7.51	0.512112	-10.26	0.14
669	7.56	0.512232	-7.92	0.14
671	7.57	0.512120	-10.11	0.14
853.3	9.32	0.512194	-8.65	0.14
861.98	9.71	0.512158	-9.35	0.14
939.6	10.39	0.512219	-8.17	0.14
1035.4	11.24	0.512344	-5.74	0.28

CSF-A - core depth below seafloor; R - represents replicate analysis.

Supplementary Table 3: Water masses and their characteristics Nd isotope compositions in the modern ocean.

Water mass	ε_{Nd}	[Nd]	reference
NADW	-13.5±0.5	17.5±1	[25,26]
AABW	-8.5±0.5	25.1±1	[25,26]
PDW	-3.5±0.5	35±1	[25,26]

Supplementary References

1 Gourlan, A. T., Meynadier, L. & Allègre, C. J. Tectonically driven changes in the Indian Ocean circulation over the last 25 Ma: Neodymium isotope evidence. *Earth and Planetary Science Letters* **267**, 353-364, doi:10.1016/j.epsl.2007.11.054 (2008).

2 Martin, E. E. & Scher, H. A Nd isotopic study of southern sourced waters and Indonesian Throughflow at intermediate depths in the Cenozoic Indian Ocean. *Geochemistry, Geophysics, Geosystems* **7**, n/a-n/a, doi:10.1029/2006gc001302 (2006).

3 Le Houedec, S., Meynadier, L. & Allègre, C. J. Nd isotope systematics on ODP Sites 756 and 762 sediments reveal major volcanic, oceanic and climatic changes in South Indian Ocean over the last 35Ma. *Earth and Planetary Science Letters* **327-328**, 29-38, doi:10.1016/j.epsl.2012.01.019 (2012).

4 Goswami, V., Singh, S. K. & Bhushan, R. Impact of water mass mixing and dust deposition on Nd concentration and ε Nd of the Arabian Sea water column. *Geochimica et Cosmochimica Acta* **145**, 30-49, doi:10.1016/j.gca.2014.09.006 (2014).

5 Suresh, K., Kumar, A., Ramaswamy, V. & Prakash Babu, C. Seasonal variability in aeolian dust deposition fluxes and their mineralogical composition over the Northeastern Arabian Sea. *International Journal of Environmental Science and Technology*, doi:10.1007/s13762-021-03503-y (2021).

6 Kumar, A., Suresh, K. & Rahaman, W. Geochemical characterization of modern aeolian dust over the Northeastern Arabian Sea: Implication for dust transport in the Arabian Sea. *Science of The Total Environment* **729**, 138576, doi:https://doi.org/10.1016/j.scitotenv.2020.138576 (2020).

7 Gourlan, A. T. *et al.* Northern Hemisphere climate control of the Bengali rivers discharge during the past 4 Ma. *Quaternary Science Reviews* **29**, 2484-2498, doi:10.1016/j.quascirev.2010.05.003 (2010).

8 O'Nions, R. K., Frank, M., von Blanckenburg, F. & Ling, H. F. Secular variation of Nd and Pb isotopes in ferromanganese crusts from the Atlantic, Indian and Pacific Oceans. *Earth and Planetary Science Letters* **155**, 15-28, doi:https://doi.org/10.1016/S0012-821X(97)00207-0 (1998).

9 Frank, M., Whiteley, N., van de Flierdt, T., Reynolds, B. C. & O'Nions, K. Nd and Pb isotope evolution of deep water masses in the eastern Indian Ocean during the past 33 Myr. *Chemical Geology* **226**, 264-279, doi:https://doi.org/10.1016/j.chemgeo.2005.09.024 (2006).

10 Lathika, N. *et al.* Deep water circulation in the Arabian Sea during the last glacial cycle: Implications for paleo-redox condition, carbon sink and atmospheric CO2 variability. *Quaternary Science Reviews* **257**, 106853, doi:https://doi.org/10.1016/j.quascirev.2021.106853 (2021).

11 Clift, P. D. *et al.* Chemical weathering and erosion responses to changing monsoon climate in the Late Miocene of Southwest Asia. *Geological Magazine*, 1-17, doi:10.1017/s0016756819000608 (2019).

12 Khim, B.-K. *et al.* Variations in δ13C values of sedimentary organic matter since late Miocene time in the Indus Fan (IODP Site 1457) of the eastern Arabian Sea. *Geological Magazine*, 1-10, doi:10.1017/s0016756818000870 (2019).

13 Routledge, C. M. *et al.* A revised chronostratigraphic framework for International Ocean Discovery Program Expedition 355 sites in Laxmi Basin, eastern Arabian Sea. *Geological Magazine*, 1-18, doi:10.1017/s0016756819000104 (2019).

14 Pandey, D. K., Clift, P. D., Kulhanek, D. K. & Scientists, a. t. E. Site U1457. *Proceedings of the International Ocean Discovery Program* **355**, 1-49, doi:10.14379/iodp.proc.355.104.2016 (2016).

15 Naik, S. S., Basak, C., Goldstein, S. L., Naidu, P. D. & Naik, S. N. A 16-kyr Record of Ocean Circulation and Monsoon Intensification From the Central Bay of Bengal. *Geochemistry, Geophysics, Geosystems* **20**, 872-882, doi:10.1029/2018gc007860 (2019).

16 Huang, Y., Clemens, S. C., Liu, W., Wang, Y. & Prell, W. L. Large-scale hydrological change drove the late Miocene C4 plant expansion in the Himalayan foreland and Arabian Peninsula. *Geology* **35**, 531, doi:10.1130/g23666a.1 (2007).

17 Gupta, A. K., Yuvaraja, A., Prakasam, M., Clemens, S. C. & Velu, A. Evolution of the South Asian monsoon wind system since the late Middle Miocene. *Palaeogeography, Palaeoclimatology, Palaeoecology* **438**, 160-167, doi:10.1016/j.palaeo.2015.08.006 (2015).

18 Gupta, A. K., Singh, R. K., Joseph, S. & Thomas, E. Indian Ocean high-productivity event (10–8 Ma): Linked to global cooling or to the initiation of the Indian monsoons? *Geology* **32**, 753, doi:10.1130/g20662.1 (2004).

19 Bolton, C. T. *et al.* Secular and orbital-scale variability of equatorial Indian Ocean summer monsoon winds during the late Miocene. *Clim. Past* **18**, 713-738, doi:10.5194/cp-18-713-2022 (2022).

20 Westerhold, T. *et al.* An astronomically dated record of Earth's climate and its predictability over the last 66 million years. *Science* **369**, 1383, doi:10.1126/science.aba6853 (2020).

21 Rohling, E. J. *et al.* Sea level and deep-sea temperature reconstructions suggest quasi-stable states and critical transitions over the past 40 million years. **7**, eabf5326, doi:doi:10.1126/sciadv.abf5326 (2021).

22 Reynolds, B. C., Frank, M. & O'Nions, R. K. Nd- and Pb-isotope time series from Atlantic ferromanganese crusts: implications for changes in provenance and paleocirculation over the last 8 Myr. *Earth and Planetary Science Letters* **173**, 381-396, doi:https://doi.org/10.1016/S0012-821X(99)00243-5 (1999).

23 Thomas, D. J. & Via, R. K. Neogene evolution of Atlantic thermohaline circulation: Perspective from Walvis Ridge, southeastern Atlantic Ocean. *Paleoceanography* **22**, doi:10.1029/2006pa001297 (2007).

24 Rahlf, P. *et al.* Tracing water mass mixing and continental inputs in the southeastern Atlantic Ocean with dissolved neodymium isotopes. *Earth and Planetary Science Letters* **530**, 115944, doi:https://doi.org/10.1016/j.epsl.2019.115944 (2020).

25 Howe, J. N. *et al.* North Atlantic Deep Water Production during the Last Glacial Maximum. *Nature communications* **7**, 11765, doi:10.1038/ncomms11765 (2016).

26 Yu, J. *et al.* Last glacial atmospheric CO2 decline due to widespread Pacific deep-water expansion. *Nature Geoscience* **13**, 628-633, doi:10.1038/s41561-020-0610-5 (2020).
